# Serotonin Transporter Binding in the Human Brain After Pharmacological Challenge Measured Using PET and PET/MR

**DOI:** 10.3389/fnmol.2019.00172

**Published:** 2019-07-12

**Authors:** Leo R. Silberbauer, Gregor Gryglewski, Neydher Berroterán-Infante, Lucas Rischka, Thomas Vanicek, Verena Pichler, Marius Hienert, Alexander Kautzky, Cecile Philippe, Godber M. Godbersen, Chrysoula Vraka, Gregory M. James, Wolfgang Wadsak, Markus Mitterhauser, Marcus Hacker, Siegfried Kasper, Andreas Hahn, Rupert Lanzenberger

**Affiliations:** ^1^Department of Psychiatry and Psychotherapy, Medical University of Vienna, Vienna, Austria; ^2^Department of Biomedical Imaging and Image-guided Therapy, Division of Nuclear Medicine, Medical University of Vienna, Vienna, Austria; ^3^Center for Biomarker Research in Medicine, Graz, Austria; ^4^Ludwig Boltzmann Institute Applied Diagnostics, Vienna, Austria

**Keywords:** serotonin transporter, [^11^C]DASB, bolus plus infusion, PET, neuroimaging, quantification

## Abstract

**Introduction:** *In-vivo* quantification of the serotonin transporter (SERT) guided our understanding of many neuropsychiatric disorders. A recently introduced bolus plus constant infusion protocol has been shown to allow the reliable determination of SERT binding with reduced scan time. In this work, the outcomes of two methods, a bolus injection paradigm on a GE PET camera, and a bolus plus infusion paradigm on a combined Siemens PET/MR camera were compared.

**Methods:** A total of seven healthy subjects underwent paired PET and paired PET/MR scans each with intravenous double-blind application of 7.5 mg citalopram or saline in a randomized cross-over study design. While PET scans were performed according to standard protocols and non-displaceable binding potentials (BP_ND_) were calculated using the multi-linear reference tissue model, during PET/MR measurements [^11^C]DASB was applied as bolus plus constant infusion, and BP_ND_ was calculated using the steady state method and data acquired at tracer equilibrium. Occupancies were calculated as the relative decrease in BP_ND_ between saline and citalopram scans.

**Results:** During placebo scans, a mean difference in BP_ND_ of −0.08 (−11.71%) across all ROIs was found between methods. PET/MR scans resulted in higher BP_ND_ estimates than PET scans in all ROIs except the midbrain. A mean difference of −0.19 (−109.40%) across all ROIs between methods was observed for citalopram scans. PET/MR scans resulted in higher BP_ND_ estimates than PET scans in all ROIs. For occupancy, a mean difference of 23.12% (21.91%) was observed across all ROIs. PET/MR scans resulted in lower occupancy compared to PET scans in all ROIs except the temporal cortex. While for placebo, BP_ND_ of high-binding regions (thalamus and striatum) exhibited moderate reliability (ICC = 0.66), during citalopram scans ICC decreased (0.36–0.46). However, reliability for occupancy remained high (0.57–0.82).

**Conclusion:** Here, we demonstrated the feasibility of reliable and non-invasive SERT quantification using a [^11^C]DASB bolus plus constant infusion protocol at a hybrid PET/MR scanner, which might facilitate future pharmacological imaging studies. Highest agreement with established methods for quantification of occupancy and SERT BP_ND_ at baseline was observed in subcortical high-binding regions.

## Introduction

The serotonin transporter (SERT) is the molecular target of selective serotonin reuptake inhibitors (SSRIs) that are used as the predominant first-line treatment for major depressive disorder (MDD). *In-vivo* quantification of the SERT guided our understanding of the pathophysiology of depression and many other neuropsychiatric disorders (Spies et al., 2015). It has been an exciting journey from the first human trials with [^123^I]β-CIT (Laruelle et al., 1993; Farde et al., 1994) to the demonstration of reduced SERT binding in depressed patients suffering from seasonal affective disorder, which was first published as an abstract in 1997 and later as full publication by Willeit et al. (2000) from our department. While the SERT has become one of the most extensively investigated targets in the field of neuropsychiatric disorders, it has yet to be established as a clinically relevant neuroimaging marker. However, recent meta-analytic approaches strongly support the hypothesis of reduced SERT availability in depression and indicate that many previous studies have been underpowered to detect significant changes in molecular imaging markers (Gryglewski et al., 2014). Acquisition of stable SERT imaging measures strongly relies on methodological improvements in imaging modalities and the synthesis of suitable radiopharmaceuticals. Positron emission tomography (PET) along with the application of highly specific and sensitive radiotracers offers the unique ability to measure protein expression *in-vivo* at good spatial resolution and high signal to noise ratio and, thus, has taken molecular imaging to a next level, when compared to previous technologies, such as single photon emission computed tomography (SPECT) (Rahmim and Zaidi, 2008). While several radioligands have been developed for *in-vivo* SERT imaging, applicability in clinical research of most of these radioligands was limited due to insufficient specific to non-specific binding (Paterson et al., 2013). However, calculation of binding potentials (BP_F_, BP_P_) using [^11^C]*N,N*-dimethyl-2-(2-amino-4-cyanophenylthio)benzylamine [(^11^C)DASB], a highly specific PET tracer with low non-specific binding and favorable brain kinetics, and kinetic modeling using one or two tissue compartmental models is considered state-of-the-art in the quantification of the cerebral SERT *in-vivo* (Ginovart et al., 2001). In order to overcome the rather invasive and logistically complex procedure of arterial blood sampling that is required for determination of BP_F_ and BP_P_ by means of kinetic modeling, reference tissue methods with cerebellar gray matter as reference region that is devoid of SERT have been established. Among these, calculation of the non-displaceable binding potential (BP_ND_) using the multilinear reference tissue model (MRTM2) has been shown to yield the best trade-off between errors in BP_ND_ estimates and clinical applicability (Ichise et al., 2003). Pseudoequilibrium, i.e., a transient constant tissue ratio between receptor rich regions and a reference region, is assumed to be reached after bolus injection of the tracer, however, this approach has several limitations (Ichise et al., 2001). Since sufficient equilibrium between all compartments may not be reached in bolus injection experiment, quantification of SERT is dependent on kinetic modeling which is sensitive to errors in modeling assumptions, and outlying data points. Further, given the kinetics of [^11^C]DASB, PET data acquisition for at least 90 min is required in order to obtain accurate measures of SERT availability in high density regions. Moreover, determination of SERT occupancy requires two separate scans in order to obtain pre- and post-treatment data. In a different approach equilibrium can be reached when reversibly binding radiotracers are delivered as bolus plus constant infusion (Lassen, 1992; Carson et al., 1993). This allows the calculation of the total volume of distribution (V_T_) as ratio of radioactive tracer in brain tissue to that in plasma. Using the cerebellum as a reference region, BP_ND_ can subsequently be calculated by substracting cerebellar V_T_ from the region of interest (Innis et al., 2007).

The recent introduction of hybrid PET/MR imaging systems intrigued the neuroscientific community since it enables the correlation of simultaneously acquired functional and molecular imaging measures. Thus, PET/MR bears the potential to broaden our understanding of neuropsychiatric disorders. In particular, the combination of radioligand bolus plus constant infusion and hybrid PET/MR imaging provides an integrated approach to investigate human brain function. While the calculation of BP_ND_ using [^11^C]DASB PET imaging is considered state of the art in *in-vivo* SERT quantification (Ginovart et al., 2001) and comparability of data acquisition and preprocessing has recently been reviewed across 21 PET centers, SERT binding data obtained using hybrid PET/MR imaging and tracer bolus plus constant infusion paradigms is scarce (Norgaard et al., 2019). Since reliable estimation of SERT binding measured at PET/MR imaging systems using bolus plus infusion protocols is an essential prerequisite for integrated pharmacological research comprising molecular and functional imaging measures, respectively, we aimed to validate a novel PET/MR imaging system using a promising tracer bolus plus constant infusion protocol.

## Materials and Methods

### Subjects

Subjects were recruited via postings at public places in the General Hospital of Vienna (AKH) and advertisements at local supermarkets. Mental and physical health was confirmed by medical history, physical examination, structured clinical interview for DSM-IV (SCID I), electrocardiogram, and routine laboratory parameters. Urine drug screening and pregnancy tests were obtained before each scan.

15 healthy volunteers were examined using PET and PET/MR scans each with the highly selective SERT radioligand [^11^C]DASB.

Among these, 12 subjects underwent PET scans with application of [^11^C]DASB as bolus after double blind infusion of citalopram or saline solution. While, placebo scans were successfully performed in 12 subjects, 2 dropped out of the study without completing the verum scan. In one subject kinetic modeling was not possible due to failed convergence of the algorithm. Moreover, one subject was excluded from data analysis due to failed infusion of the study medication. PET scans from the remaining 8 subjects were separated by a mean of 17 ± 15 days.

Hybrid PET/MR scans using [^11^C]DASB bolus plus constant infusion were performed twice in 12 subjects. Double-blind infusion of the study medication was performed during each PET/MR scan. One of these subjects was excluded from analysis due to failed infusion of the study medication. PET/MR scans from the remaining 11 subjects were separated by a mean of 27 ± 20 days.

Ultimately, data from 7 subjects that successfully completed both two PET and two PET/MR scans was included in reliability analyses.

### Radioligand Preparation and Application

[^11^C]DASB [(^11^C)*N,N*-dimethyl-2-(2-amino-4-cyanophenyl thio)benzylamine] was synthesized as described previously (Haeusler et al., 2009) at the Department of Biomedical Imaging and Image-guided Therapy, Division of Nuclear Medicine, Medical University of Vienna. Quality control was performed as described previously including measurement of radiochemical and chemical purity using HPLC, pH, isotonicity, radionuclidic purity and residual solvents using gas chromatography (Gryglewski et al., 2017). Sterility and endotoxines were controlled according to regulations for radiopharmaceutical preparations laid down in the European Pharmacopeia.

Mean molar activity at the end of synthesis was 107 ± 76 GBq/μmol. The minimum molar activity used was 12 GBq/μmol during a PET scan.

For [^11^C]DASB bolus PET scans, the mean injected activity was 345.24 ± 40.95 MBq (range: 270.9–447.7). For [^11^C]DASB bolus plus constant infusion PET/MR scans, the mean injected activity was 718.3 ± 82.45 MBq (range: 592–867). Thus, study participants received a mean effective dose of ca. 13.4 mSv during participation including low-dose CT and two transmission scans (Lu et al., 2004). For PET scans with bolus application, a dosage of 4.9 ± 0.2 MBq/kg was applied. For PET/MR scans with bolus plus constant infusion, a dosage of 10.68 ± 0.74 MBq/kg was prepared and diluted with sodium chloride to allow for application by an automated syringe pump.

A total of 20 milliliters were applied as bolus over 60 s, the rest was applied at a constant rate over the remaining scan time. Previously, the optimal ratio between bolus and infusion to attain equilibrium in striatum and thalamus rapidly was determined to be K_bol_ = 175 ± 35 min *in silico*. However, our results demonstrated that a slightly lower K_bol_ might be superior for these regions (Gryglewski et al., 2017). Therefore, K_bol_ was adapted throughout the study in order to empirically determine the optimal value for rapid equilibration in the thalamus and striatum. Once assigned to subjects infusion rates did not change during the investigation. Therefore, infusion rates were adapted over the course of the trial. Infusion rates were 6.7 and 7.5 ml/h for 3 subjects each and 7.1 ml/h for 6 subjects, resulting in a K_bol_ of 179, 160 and 169 min, respectively.

### Study Drug Preparation and Application

The double-blind study medication was prepared by the hospital pharmacy of the AKH Vienna (General Hospital Vienna) and provided in standard syringes containing either 7.5 mg citalopram (Seropram^®^ Lundbeck A/S) diluted in 0.9% saline solution, or placebo (55 ml of 0.9% saline solution). In a randomized cross-over study design, the double-blind application of the study medication was performed 30 min before PET scans and 60 min after initiation of PET/MR scans. While peak concentrations in plasma occur immediately after intravenous application of citalopram and rapidly decrease due to tissue distribution (Gryglewski et al., 2019), the subsequent clearance is low and occurs at a half-life of approximately 24 h (Sogaard et al., 2005). Therefore, citalopram concentration at SERT sites in the brain is expected to be relatively stable over the time course of both PET and PET/MR experiments. The administration of the study drug was performed using a PET/MR compatible fully automated infusion system (Syramed^®^μSP6000, arcomed ag, Regensdorf, Switzerland).

### Data Acquisition

Positron emission tomography scans were performed at the Department of Biomedical Imaging and Image-guided Therapy, Medical University of Vienna, using a GE Advance full-ring scanner (General Electric Medical Systems, Milwaukee, WI, United States) in 3D mode, as described previously (Lanzenberger et al., 2012). A 5 min transmission scan was performed using retractable ^68^Ge rod sources for tissue attenuation correction. Subsequently, data acquisition started simultaneously with a bolus injection of [^11^C]DASB measuring brain radioactivity in a series of 51 consecutive time frames (12 × 5 s, 6 × 10 s, 3 × 20 s, 6 × 30 s, 9 × 1 min, 15 × 5 min) over 90 min.

PET/MR scans were performed using a 3 Tesla SIEMENS mMR Biograph scanner (Siemens Medical, Erlangen, Germany) at the Department of Biomedical Imaging and Image-guided Therapy, Medical University of Vienna. Application of [^11^C]DASB was initiated simultaneously with acquisition of PET data in listmode for 145 min. Attenuation correction was performed with a separate low-dose CT scan (Siemens Biograph TruePoint PET/CT) since CT derived attenuation correction is considered the gold standard procedure for PET/MR data and MR derived attenuation correction is accompanied by high inaccuracies (Ladefoged et al., 2017). The CT was spatially co-registered to the T_1_-weighted image with SPM12 and scaled bi-linearly to obtain an attenuation map (Carney et al., 2006; Ladefoged et al., 2017).

### Preprocessing and Regions of Interest

Reconstruction of GE PET data (voxel size = 3.13 mm × 3.13 mm × 4.25 mm) into 35 transaxial sections (128 × 128) was performed as described previously (Gryglewski et al., 2017). List-mode PET/MR data (voxel size = 2.09 mm × 2.09 mm × 2.03 mm, image matrix 344 × 344 × 127) was reconstructed with an ordinary Poisson ordered subset expectation maximization algorithm (OP-OSEM, 3 iterations, 21 subsets). Data was corrected for motion and co-registered to T_1_-weighted MRI data that was normalized to montreal neurological institute (MNI) space using SPM12 (Wellcome Trust Center for Neuroimaging, London, United Kingdom^1^).

Using the automated anatomical labeling (AAL) atlas time-activity curves were extracted from regions of interest (ROIs) with high SERT binding (Savli et al., 2012) [thalamus, striatum, putamen, caudate nucleus, midbrain, amygdala, olfactory cortex, and anterior cingulate cortex (ACC)] (Figure 2). As described previously, a ROI for cerebellar gray matter excluding vermis and the venous sinus was delineated (Lanzenberger et al., 2012). Moreover, ROIs for the frontal, temporal, parietal and occipital cortex were created by combining AAL ROIs located in the respective lobes. ROIs from left and right hemispheres were averaged where applicable.

### SERT Quantification

For PET bolus scans quantification of SERT non-displaceable BP_ND_ was carried out using the multilinear reference tissue model (MRTM2) (Ichise et al., 2003) in PMOD 3.7. Here, cerebellar gray matter was used as reference region, the striatum as high binding region. The MRTM model was used for initial estimation of individual k’_2_ values fixed to cerebellar gray matter (Hahn et al., 2014) that were subsequently inserted into MRTM2 to compute BP_ND_. T^*^ was individually fitted for each region in PMOD. The lower and upper threshold values were set at 0 and 10, respectively. Thus, BP_ND_ below zero were set to zero and BPND above ten were set to ten.

**FIGURE 1:**
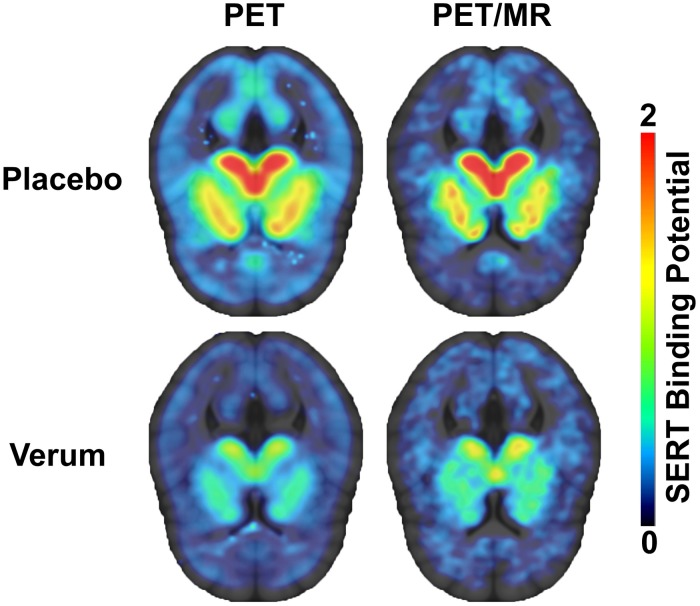
Serotonin transporter binding potentials (BP_ND_) obtained using [^11^C]DASB bolus PET scans (left) and [^11^C]DASB bolus plus constant infusion PET/MR scans (right) during placebo (top row) and verum condition (bottom row) are displayed in transversal planes.

For [^11^C]DASB bolus plus infusion PET/MR scans distribution volumes in ROIs (C_T_) and the reference region (C_ND_) were calculated by averaging ROI activity at tracer equilibrium using 4 frames à 5 min obtained between 100 and 120 min after start of radioligand application. Subsequently, BP_ND_ was calculated as follows: BP_ND_ = (C_T_ – C_ND_)/C_ND_ (Figure 1). Occupancies were calculated as the relative decrease in BP_ND_ between saline and citalopram scans.

**FIGURE 2:**
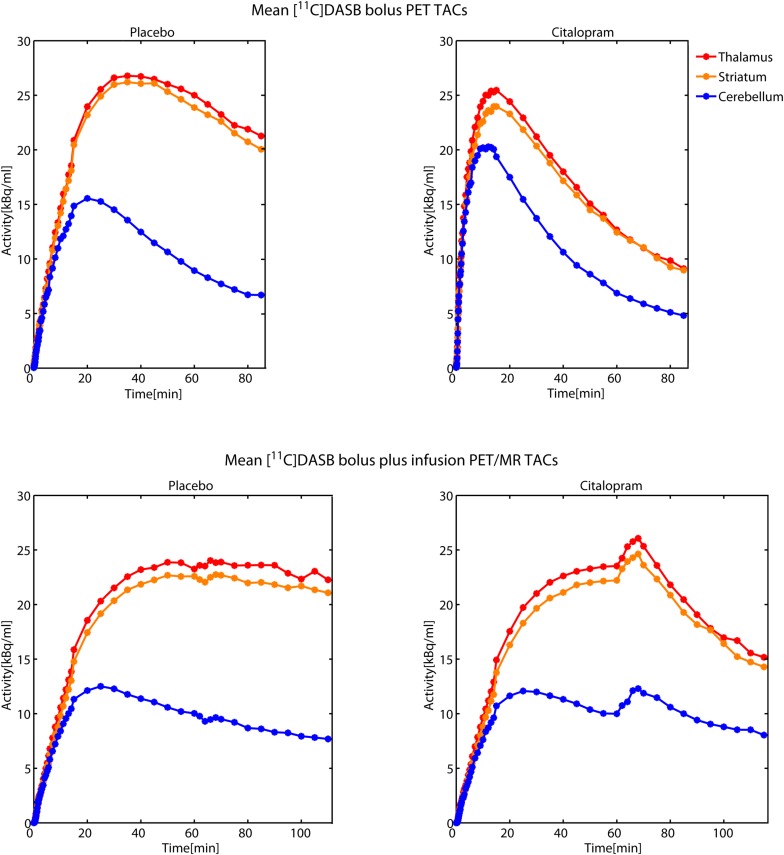
Time activity curves (TACs) from [^11^C]DASB bolus PET scans (upper row) and [^11^C]DASB bolus plus constant infusion PET/MR scans (bottom row) during placebo (left) and citalopram condition (right) are averaged across 7 subjects each. TACs from striatum, thalamus and the cerebellum are illustrated in red, orange, and blue color, respectively. Double-blind application of the pharmacological challenge was performed 60 min after initiation of [^11^C]DASB bolus plus constant infusion PET/MR scans. Transient increases in tissue activity during the citalopram scans (bottom right) might be caused by peripheral displacement of the tracer from SERT rich tissues, such as the lungs.

### Inter-Method Reliability Analysis

Binding potentials of placebo and verum scans (BP_ND_-_*PL*_ and BP_*ND–VER*_), respectively, obtained from [^11^C]DASB bolus PET scans were compared to those obtained from [^11^C]DASB bolus plus constant infusion PET/MR scans. Moreover, resulting SERT occupancy data was compared between methods. Mean difference and intraclass correlation coefficients (ICC) were calculated in R 3.3.0 (R Foundation for Statistical Computing, Vienna, Austria^2^). Mean difference was calculated as the difference between scans divided by the value of the bolus plus constant infusion measurement. The variability was calculated as the standard deviation of the mean difference. The ICC was calculated as follows:

$$\rho =\frac{\left(\mathrm{MSB}-\mathrm{MSE}\right)}{\left[\mathrm{MSB}+\left(k-1\right)\times \mathrm{MSE}\right]}$$

MSB being the mean square between, MSE being the mean square error and k the number of measurements per subjects, that is 2. ICCs are reported according to Koo and Li (2016): ICC values less than 0.5 are indicative of poor reliability, values between 0.5 and 0.75 indicate moderate, values between 0.75 and 0.9 indicate good, and values greater than 0.9 indicate excellent reliability. Moreover, Bland-Altman plots were constructed in R to assess agreement between methods.

## Results

### Comparison of BP_ND_ Between [^11^C]DASB PET and PET/MR Scans

**FIGURE 3:**
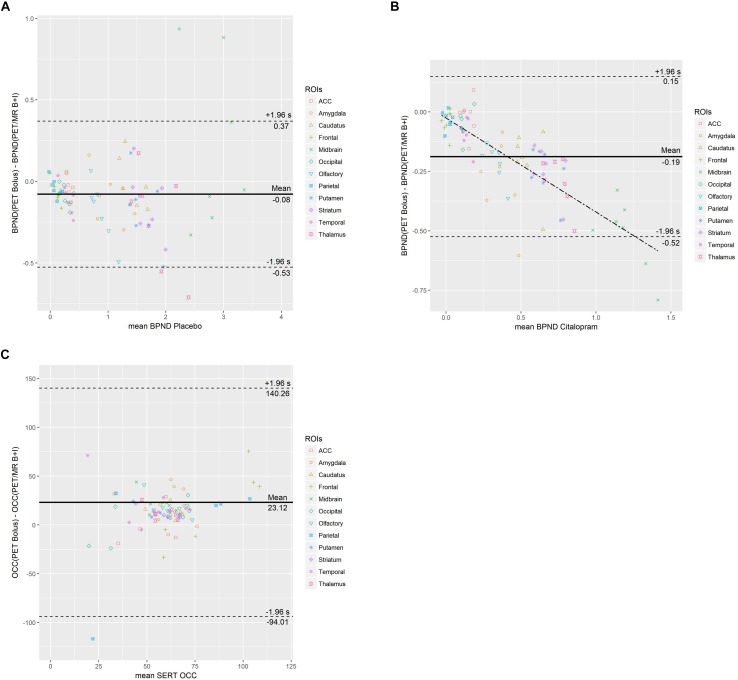
Bland-Altman plots of difference between PET and PET/MR imaging procedures vs. the mean of the two measurements are displayed for BP_ND_ during placebo **(A)** and citalopram condition **(B)** as well as SERT occupancy **(C)**. The mean difference (bias) is represented by the parallel solid line. Limits of agreement are represented from –1.96^*^ SD to +1.96^*^ SD by dotted lines. In **(B)** the dash-dotted line represents the regression line (y = –0.3953× – 0.0258). In **(C)** data points with a mean occupancy ≤120% are shown.

Inter-method reliability of BP_ND_ obtained from [^11^C]DASB bolus PET scans and [^11^C]DASB bolus plus constant infusion PET/MR scans are displayed in Tables 1, 2 for placebo and citalopram scans, respectively. Bland-Altman plots of difference between PET and PET/MR imaging procedures vs. the mean of the two measurements are displayed for BP_ND_ during placebo (a) and citalopram condition (b) in Figure 3.

**TABLE 1:** Results of inter-method reliability analysis comparing binding potentials (BP_ND_) obtained from [^11^C]DASB bolus PET scans using the multilinear reference tissue model (MRTM2) with BP_ND_ obtained from [^11^C]DASB bolus plus constant infusion PET/MR scans using the equilibrium method during placebo challenge.

**Region of interest**	**BP_ND_ PET/MR**	**SD**	**BP_ND_ PET**	**SD**	**Mean difference (%)**	**SD**	**MSB**	**MSE**	**ICC (ρ)**
Frontal cortex	0.14	0.10	0.08	0.04	−60.83	0.48	0.010	0.002	0.63
Occipital cortex	0.31	0.12	0.22	0.08	−26.34	0.17	0.019	0.002	0.79
Parietal cortex	0.11	0.10	0.07	0.06	−51.15	0.35	0.013	0.002	0.78
Temporal cortex	0.35	0.14	0.25	0.06	−21.21	0.26	0.019	0.004	0.62
Anterior cingulate cortex	0.35	0.07	0.31	0.03	−7.77	0.22	0.003	0.003	0.08
Amygdala	1.10	0.34	1.00	0.25	−6.58	0.17	0.165	0.012	0.86
Olfactory cortex	0.96	0.26	0.78	0.09	−15.73	0.14	0.062	0.016	0.58
Caudate nucleus	1.44	0.25	1.41	0.16	−0.13	0.12	0.075	0.013	0.71
Putamen	1.69	0.31	1.52	0.19	−8.83	0.12	0.109	0.023	0.65
Thalamus	1.93	0.48	1.70	0.29	−9.85	0.13	0.263	0.048	0.69
Striatum	1.73	0.30	1.61	0.19	−5.98	0.11	0.107	0.020	0.69
Midbrain	2.71	0.50	2.92	0.44	10.68	0.24	0.305	0.136	0.38

**TABLE 2:** Results of inter-method reliability analysis comparing BP_ND_ obtained from [^11^C]DASB bolus PET scans using the multilinear reference tissue model (MRTM2) with BP_ND_ obtained from [^11^C]DASB bolus plus constant infusion PET/MR scans using the equilibrium method during citalopram challenge.

**Region of interest**	**BP_ND_ PET/MR**	**SD**	**BP_ND_ PET**	**SD**	**Mean difference (%)**	**SD**	**MSB**	**MSE**	**ICC (ρ)**
Frontal cortex	0.03	0.03	−0.01	0.04	−39.49	2.07	0.001	0.001	−0.11
Occipital cortex	0.15	0.03	0.09	0.05	−36.60	0.32	0.002	0.002	0.06
Parietal cortex	0.03	0.03	0.00	0.03	−56.21	1.45	0.001	0.001	0.20
Temporal cortex	0.18	0.05	0.09	0.03	−47.12	0.22	0.001	0.002	−0.18
Anterior cingulate cortex	0.15	0.05	0.14	0.05	−3.68	0.40	0.002	0.003	−0.06
Amygdala	0.51	0.15	0.22	0.10	−54.97	0.23	0.016	0.015	0.05
Olfactory cortex	0.43	0.09	0.22	0.04	−48.09	0.08	0.007	0.003	0.40
Caudate nucleus	0.64	0.12	0.44	0.08	−30.93	0.14	0.012	0.009	0.11
Putamen	0.79	0.12	0.53	0.07	−31.46	0.09	0.014	0.005	0.44
Thalamus	0.90	0.12	0.60	0.06	−32.24	0.08	0.012	0.006	0.36
Striatum	0.79	0.12	0.55	0.07	−30.03	0.09	0.015	0.005	0.47
Midbrain	1.45	0.20	0.94	0.10	−35.09	0.06	0.040	0.012	0.55

#### Placebo Scans

The Bland-Altman plot demonstrates a mean across all ROIs between methods. No trend of mean differences across the range of mean BP_ND_ was observed for individual regions. Compared to BP_ND_ obtained from [^11^C]DASB PET/MR placebo scans, BP_ND_ obtained from [^11^C]DASB PET placebo scans resulted in a negative mean difference ranging from −60.83% to −0.13% in all ROIs except the midbrain which exhibited a positive mean difference of 10.68%. In other words, PET/MR placebo scans resulted in higher BP_ND_ estimates than PET scans in all ROIs except the midbrain. High binding regions such as the thalamus and the striatum exhibited low mean difference of −9.85 and −5.98% and an ICC of 0.69. Lower binding cortical ROIs exhibited more variable mean differences ranging from −21.21 to −60.83% and ICCs between 0.62 and 0.79. A mean difference of 10.68% for midbrain with a low ICC of 0.38 was observed.

#### Citalopram Scans

The Bland-Altman plot demonstrates a mean difference of −0.19 across all ROIs between methods. In lower binding regions, a slightly negative trend of differences proportional to the magnitude of the measurement is shown. Compared to [^11^C]DASB PET/MR citalopram scans, BP_ND_ obtained from [^11^C]DASB PET citalopram scans were lower in all investigated ROIs with a mean difference ranging from −3.68 to −56.21%. The mean difference of high binding ROIs such as the striatum and thalamus increased to −30.03 and −32.24% with ICCs of 0.47 and 0.36, respectively, during citalopram scans. However, low binding ROIs exhibited more pronounced mean differences ranging from −36.60 to −56.21% with ICCs ranging from −0.18 to 0.20 during citalopram scans. For further details, see Table 2.

### Comparison of SERT Occupancy Between [11C]DASB PET and PET/MR Scans

Inter-method reliability of SERT occupancy obtained from [^11^C]DASB PET scans and [^11^C]DASB PET/MR scans is displayed in Table 3. The Bland-Altman plot of difference between PET and PET/MR imaging procedures vs. the mean of the two measurements is displayed in Figure 3C for SERT occupancy and demonstrates a mean difference of 23.12 across all ROIs. No trend of mean differences across the range of mean occupancy is observed for individual regions.

**TABLE 3:** Results of inter-method reliability analysis comparing SERT occupancy (OCC) obtained from [^11^C]DASB bolus PET scans using the multilinear reference tissue model (MRTM2) with SERT OCC obtained from [^11^C]DASB bolus plus constant infusion PET/MR scans using the equilibrium method.

**Region of interest**	**OCC PET/MR**	**SD**	**OCC PET**	**SD**	**Mean difference (%)**	**SD**	**MSB**	**MSE**	**ICC (ρ)**
Frontal cortex	74.39	10.58	129.62	101.63	82.33	1.62	4914.872	5525.347	−0.06
Occipital cortex	48.62	16.25	53.57	30.35	7.88	0.54	977.247	208.188	0.65
Parietal cortex	68.22	23.99	146.24	170.46	145.30	2.81	14586.857	15045.049	−0.02
Temporal cortex	41.89	27.22	62.21	15.62	−42.61	1.74	674.947	309.764	0.37
Anterior cingulate cortex	52.86	20.48	54.74	17.37	23.64	0.80	513.258	207.879	0.42
Amygdala	52.61	8.43	78.07	10.04	53.19	0.40	55.212	116.635	−0.36
Olfactory cortex	52.45	13.54	71.03	6.62	44.35	0.46	167.682	59.646	0.48
Caudate nucleus	54.42	8.76	68.86	6.20	28.51	0.17	89.552	25.620	0.56
Putamen	52.34	10.82	64.38	6.73	26.75	0.23	145.719	16.746	0.79
Thalamus	51.81	9.38	63.78	5.94	26.11	0.23	97.240	26.147	0.58
Striatum	53.09	10.43	65.35	6.11	26.12	0.19	132.921	13.175	0.82
Midbrain	44.96	11.15	67.32	5.94	61.42	0.60	103.889	55.779	0.30

Compared to PET/MR scans, occupancy data obtained from PET scans resulted in a positive mean difference ranging from 7.88 to 82.33% for all ROIs but a negative mean difference of −42.61% for the temporal cortex. In other words, PET/MR scans resulted in lower occupancy compared to PET scans in all ROIs except the temporal cortex. For the thalamus and striatum, i.e., high binding regions, there was a positive mean difference of 26.11 and 26.12% and ICCs ranging from 0.58 to 0.82, respectively. For lower binding cortical ROIs there was a mean difference of up to 82.33% in the frontal cortex and ICCs ranging from −0.06 to 0.65.

### k’_2_ and SUV Estimation

Results of individual k’_2_ values of each subject obtained from [^11^C]DASB bolus PET scans and cerebellar standardized uptake values (SUVs) obtained from [^11^C]DASB bolus plus constant infusion are displayed in Table 4 for both the placebo and citalopram scans. Paired two-tailed *t*-test did not indicate significant differences between the placebo and citalopram PET (*p* = 0.5584) and PET/MR (*p* = 0.1233) scans, respectively.

**TABLE 4:** Individual k’_2_ and cerebellar SUV values are given for PET and PET/MR scans during both placebo and citalopram condition.

	**k’_2_ PET (min^–1^)**			**SUV PET/MR**		

			***t*-test**			***t*-test**

**Subject**	**Placebo**	**Citalopram**	***p* = 0.56**	**Placebo**	**Citalopram**	***p* = 0.13**
1	0.0681	0.0557		1.04	1.10	
2	0.0639	0.0661		1.09	1.16	
3	0.0607	0.2455		1.29	1.37	
4	0.0484	0.0476		1.24	1.52	
5	0.0793	0.0917		1.27	1.17	
6	0.0638	0.0617		0.82	0.99	
7	0.1009	0.0430		1.05	1.06	

*Further, *p*-values obtained from paired two-tailed *t*-test are displayed.*

## Discussion

In this study, we evaluated the reliability of SERT binding data obtained at a hybrid PET/MR scanner using an optimized [^11^C]DASB bolus plus constant infusion protocol compared to standard PET procedures with tracer bolus application.

### Binding Potentials (BP_ND_)

Quantification of SERT BP_ND_ does not require arterial blood sampling and can be calculated directly from brain data by estimation of the non-displaceable binding in the reference region, i.e., the cerebellum. However, this approach depends on the assumption that non-displaceable uptake is independent of subject groups and treatment effects (Innis et al., 2007).

Given the implication of possible bias in the calculation of SERT occupancy using the cerebellum as a reference region (Turkheimer et al., 2012), systematic differences in individual k’_2_, and SUV values between conditions were excluded. Application of MRTM2 is expected to be more stable than MRTM at high-noise levels since fewer parameters are estimated. However, this approach relies on estimation of k’_2_ fixed to that of cerebellar gray matter and k’_2_ fixed, thus, represent a possible confounder in the comparability between the PET placebo and citalopram condition. Thus, paired two-tailed *t*-tests were performed and did not demonstrate significant differences in individual k’_2_ values between conditions. Further, cerebellar standardized uptake values (SUV) were compared between the PET/MR placebo and citalopram condition in order to assess differences in reference tissue activity between conditions corrected for injected dose. Similarly, paired two-tailed *t*-test did not indicate significant differences.

Binding potentials obtained during placebo condition demonstrated moderate to good inter-method reliability of ICC 0.64 – 0.86 and a small negative mean difference <10% for most subcortical high-binding regions except the midbrain. Among cortical regions, the ACC demonstrated a small negative mean difference of −7.78% but poor inter-method reliability (ICC 0.08). The olfactory cortex, however, showed moderate negative mean difference (−15.73%) and moderate inter-method reliability (ICC 0.58). The remaining low-binding cortical regions suffered from high mean difference (ranging from 21.2 to 60.83%), however, inter-method reliability remained moderate to good with ICCs ranging from 0.62 to 0.79.

During citalopram application BP_ND_ suffered from high mean difference (ranging from 3.68% to 56.20%) and poor inter-method reliability with ICCs ranging from −0.19 to a maximum of 0.47. In short, non-invasive quantification of SERT BP_ND_ using a hybrid PET/MR scanner and [^11^C]DASB bolus plus constant infusion is reliable in high-binding subcortical regions during placebo condition. Inter-method reliability for citalopram condition might have been mitigated by temporal differences in application of the study medication, the selected time frame for estimation of the equilibrium and the overall decrease in specific binding.

In previously published test-retest analyses of [^11^C]DASB using reference tissue models and kinetic modeling, respectively, similar to somewhat stronger reliability was reported in receptor-rich regions (Frankle et al., 2006; Kim et al., 2006). While good inter-method reliability of [^11^C]DASB bolus plus constant infusion with standard bolus procedures has been previously demonstrated by our group, however, when interpreting our results it must be kept in mind that here we compared two different scanners using two different methodological approaches (Gryglewski et al., 2017).

### Occupancy

Quantification of SERT occupancy enables the determination of drug induced changes in receptor availability and thus is considered as a surrogate for the molecular mechanism of action of SSRIs. SERT occupancy indicates the relative decrease of [^11^C]DASB BP_ND_ after pharmacological SSRI challenge and thus, reliability is directly reflected by the inter-method reliability of BP_ND_.

During continuous oral SSRI treatment SERT occupancy of approximately 80% has repeatedly been demonstrated (Meyer et al., 2001, 2004). Moreover, increases in SERT occupancy over time during prolonged oral administration were observed when compared to acute SSRI intake (Baldinger et al., 2014). However, SERT occupancy data presented in this study is in line with results obtained in a previous randomized controlled trial using a single intravenous infusion of citalopram (Hinz et al., 2008). Given the long half-life of citalopram (24 h) advanced metabolism after application of the study medication 30 min before PET scans is unlikely to account for significant differences in occupancy compared to PET/MR measurements.

The mostly negative mean difference in BP_ND_ during placebo and verum scans, respectively, translated into a positive mean difference in occupancy for most regions. In larger high-density regions (caudate nucleus, putamen, thalamus and striatum) we demonstrate a maximum mean difference of 28.5% with moderate to good inter-method reliability of ICC 0.56 – 0.82. Lower-binding cortical regions suffered from high mean difference of up to 145.3% and poor inter-method reliability.

In summary, SERT occupancy can reliably be obtained using a hybrid PET/MR scanner and [^11^C]DASB bolus plus constant infusion in large high-binding subcortical regions despite relatively high mean difference and low ICCs of BP_ND_ obtained during citalopram condition. These results suggest that using this novel approach low mean difference and good inter-method reliability during placebo condition facilitate reliable estimation of SERT occupancy even in the presence of more pronounced mean difference during the citalopram condition. While high inter-method reliability as indicated by high ICC values indicates reproducibility of SERT binding across scanners, the observed methodological mean difference is corrigible by a linear factor.

### Limitations and Implications

Several limitations should be discussed. First, the sample size of this investigation is small. While initially 15 subjects were enrolled in this study, ultimately data from 7 subjects who successfully completed both two PET and PET/MR scans could be included in reliability analyses. This yet again demonstrates the sophisticated process of PET neuroimaging.

As evident from TACs in Figure 2, calculation of BP_ND_ using frames between 100 and 120 min might not be ideal for the citalopram condition and could contribute to low inter-method reliability of BP_ND_ and underestimation of occupancy with the bolus plus constant infusion method. Thus, we encourage future investigations to obtain a more robust equilibrium by measuring at later time points after drug application.

In addition, heterogenous bolus/infusion ratios were applied in this study. While a K_bol_ of 160 min has proven superior for rapid SERT quantification in high-binding midbrain regions using [^11^C]DASB, here K_bol_ varied from 160 to 179 min (Gryglewski et al., 2017). However, compared to published protocols in the literature and theoretical predictions we applied a beneficial bolus/infusion ratio for SERT quantification in these regions (Carson et al., 1993; Albin et al., 2008).

Furthermore, we compared two different methods using two different scanners. While kinetic modeling using a bolus application of [^11^C]DASB is considered state-of-the-art for SERT quantification (Ginovart et al., 2001), in the PET/MR setting several caveats using this standard protocol should be considered. First, kinetic modeling requires continuous arterial blood sampling, a procedure that is very likely to interfere with MR data acquisition, especially when it comes to sophisticated functional MRI sequences. Second, this approach necessitates acquisition of dynamic PET data for at least 90 min in order to obtain reliable measures of SERT density in high-binding regions. In contrast, using a bolus plus infusion protocol valid estimates of SERT binding can be obtained using a significantly reduced scan time (Gryglewski et al., 2017). Thus, the radiotracer bolus plus constant infusion setup might aid to overcome restricted scan time at highly demanded hybrid PET/MR scanners.

In this investigation, bolus plus constant infusion PET/MR scanning procedures were clearly associated with higher burden for study participants. First, this is evident from longer scan duration, confined space conditions and high noise level within the PET/MR scanner. Second, bolus plus constant infusion PET/MR imaging was associated with higher radiation burden. While transmission scans using the ^68^Ge rod sources and the separate low-dose CT are comparable in radiation exposure, given the decay half-life of 20 min of [^11^C]DASB a higher amount of injected activity is required to maintain sufficient activity during the infusion protocol. While estimation of occupancy within one measurement is a promising approach that might also contribute to decrease overall radiation exposure and scan time, it is still in the development stage. Future studies aiming to obtain SERT occupancy within one measurement need to correct for bias that results from divergent equilibration before and after pharmacological challenge as previously exemplified by data from our group in which SERT binding varied across time during placebo scans (Gryglewski et al., 2017).

With a spatial resolution of 4.3 mm full-width at half maximum (FWHM) spatial resolution was equal in both scanners. However, co-registration of simultaneously acquired data in the PET/MR might account for favorable spatial agreement during co-registration.

Along with the potential to integrate functional and molecular imaging measures, the applied bolus plus infusion protocol appears to be tailor-made for the purpose of hybrid PET/MR imaging.

## Conclusion

In conclusion, we demonstrated the applicability of non-invasive SERT quantification in subcortical high-binding regions using a [^11^C]DASB bolus plus constant infusion protocol at a hybrid PET/MR scanner. While moderate to good inter-method reliability was obtained for BP_ND_ during placebo, BP_ND_ obtained during citalopram administration suffered from poor inter-method reliability. However, SERT occupancy of citalopram could reliably be obtained in large subcortical high-binding regions. While the selection of either method should depend on the research question under investigation, the bolus plus constant infusion protocol along with hybrid PET/MR imaging is the method of choice in the multimodal investigation of neuropsychiatric disorders. This approach will help to decrease scan time in vulnerable patient populations and further allows for the investigation of immediate drug effects. We propose that this [^11^C]DASB bolus plus constant infusion protocol using hybrid PET/MR imaging will broaden our understanding of drug effects on human brain structure and function.

## Data Availability

All datasets generated for this study are included in the manuscript and/or the supplementary files.

## Ethics Statement

All participants provided written informed consent. All study-related procedures were reviewed and approved by the Ethics Committee of the Medical University of Vienna and performed according to the Declaration of Helsinki.

## Author Contributions

LS, NB-I, LR, VP, CV, and CP collected and analyzed the data. LS, GGR, TV, MHi, GGO, and AK provided the medical support. LS wrote the manuscript. GG conceived the study under supervision of RL and involved in data processing and analysis. GG, GMG, LR, VP, CV, TV, CP, GJ, and AH carefully revised the manuscript. TV, MHi, GG, GMG, AK, and GJ collected the data. WW, MM, and MHa conceived the study and analyzed the data. MHa and SK were the medical supervisors of the study. AH made substantial contribution to the study design and data analysis. RL supervised and investigated the study.

## Conflict of Interest Statement

SK received the grants/research support, consulting fees, and/or honoraria within the last three years from the Angelini, AOP Orphan Pharmaceuticals AG, Celegne GmbH, Eli Lilly, Janssen-Cilag Pharma GmbH, KRKA-Pharma, Lundbeck A/S, Mundipharma, Neuraxpharm, Pfizer, Sanofi, Schwabe, Servier, Shire, Sumitomo Dainippon Pharma Co. Ltd., and Takeda. RL received the conference speaker honorarium within the last three years from the Shire and research support from the Siemens Healthcare. MHa received the consulting fees and/or honoraria from the Bayer Healthcare BMS, Eli Lilly, EZAG, GE Healthcare, Ipsen, ITM, Janssen, Roche, and Siemens Healthineers. WW declares to having received speaker honoraria from the GE Healthcare and research grants from the Ipsen Pharma, Eckert-Ziegler AG, Scintomics, and ITG; and working as a part time employee of CBmed Ltd. (Center for Biomarker Research in Medicine, Graz, Austria). The remaining authors declare that the research was conducted in the absence of any commercial or financial relationships that could be construed as a potential conflict of interest.

## Footnotes

1 http://www.fil.ion.ucl.ac.uk/spm/

2 www.R-project.org
